# Long-term renal outcome in methylmalonic acidemia in adolescents and adults

**DOI:** 10.1186/s13023-021-01851-z

**Published:** 2021-05-13

**Authors:** Myriam Dao, Jean-Baptiste Arnoux, Frank Bienaimé, Anaïs Brassier, François Brazier, Jean-François Benoist, Clément Pontoizeau, Chris Ottolenghi, Pauline Krug, Olivia Boyer, Pascale de Lonlay, Aude Servais

**Affiliations:** 1 Adult Nephrology and Transplantation Department, Hôpital Necker Enfants Malades, APHP, 149 rue de Sèvres, 75015 Paris, France; 2 Reference Center of Inherited Metabolic Diseases (MAMEA and MetabERN), Hôpital Necker-Enfants Malades, APHP, 149 rue de Sèvres, 75015 Paris, France; 3 Department of Physiology, Hôpital Necker Enfants Malades, APHP, 149 rue de Sèvres, 75015 Paris, France; 4 Biochemistry Department, Hôpital Necker Enfants Malades, APHP, 149 rue de Sèvres, 75015 Paris, France; 5 Pediatric Nephrology Department, Hôpital Necker Enfants Malades, APHP, 149 rue de Sèvres, 75015 Paris, France

**Keywords:** Methylmalonic acidemia, Chronic kidney disease, Measured glomerular filtration rate, Estimated glomerular filtration rate, Tubulopathy

## Abstract

**Background:**

Chronic kidney disease (CKD) is one of the main long-term prognosis factors in methylmalonic acidemia (MMA), a rare disease of propionate catabolism. Our objective was to precisely address the clinical and biological characteristics of long-term CKD in MMA adolescent and adult patients.

**Patients and methods:**

In this retrospective study, we included MMA patients older than 13 years who had not received kidney and/or liver transplantation. We explored tubular functions, with special attention to proximal tubular function. We measured glomerular filtration rate (mGFR) by iohexol clearance and compared it to estimated glomerular filtration rate (eGFR) by Schwartz formula and CKD-EPI.

**Results:**

Thirteen patients were included (M/F = 5/8). Median age was 24 years (13 to 32). Median mGFR was 57 mL/min/1.73 m^2^ (23.3 to 105 mL/min/1.73 m^2^). Ten out of 13 patients had mGFR below 90 mL/min/1.73 m^2^. No patient had significant glomerular proteinuria. No patient had complete Fanconi syndrome. Only one patient had biological signs suggestive of incomplete proximal tubulopathy. Four out of 13 patients had isolated potassium loss, related to a non-reabsorbable anion effect of urinary methylmalonate. Both Schwartz formula and CKD-EPI significantly overestimated GFR. Bias were respectively 16 ± 15 mL/min/1.73 m^2^ and 37 ± 22 mL/min/1.73 m^2^.

**Conclusion:**

CKD is a common complication of the MMA. Usual equations overestimate GFR. Therefore, mGFR should be performed to inform therapeutic decisions such as dialysis and/or transplantation. Mild evidence of proximal tubular dysfunction was found in only one patient, suggesting that other mechanisms are involved.

**Supplementary Information:**

The online version contains supplementary material available at 10.1186/s13023-021-01851-z.

## Background

Methylmalonic acidemia (MMA) is a rare and severe inborn disease of propionate catabolism [1], caused by a defect in the mitochondrial methylmalonyl-CoA mutase (MCM). MCM isomerises L-methylmalonyl-CoA into succinyl-CoA which enters the Krebs cycle (Additional file 1: Fig. S1). MCM deficiencies are due to mutations in the *MUT* gene, encoding MCM, or to mutations in *MMAA* (CblA), *MMAB* (CblB) and occasionally *MMADHC* (CblD) genes, involved in the metabolism of its cofactor adenosylcobalamin [2]. According to residual MCM activity, *MUT* mutations are called mut^0^ for indetectable residual activity or mut^−^ for low to moderate residual activity responsive to high concentrations of adenosylcobalamin [3]. MMA usually presents as acute metabolic distress at birth, when MCM deficiency is complete, or in childhood [4]. Despite several therapeutic improvements in the past 20 years, the overall outcome of patients with MMA remains unsatisfactory [1, 4–6]. Long-term prognosis is worsened by chronic organ damage: neurological impairment and intellectual deficiency [5, 7–10], chronic kidney disease (CKD) [4, 5, 9, 11–16], optic neuropathy [17, 18], chronic pancreatitis [7, 9, 19] and osteopenia [4].

CKD is a common complication of MMA [4, 5, 9, 11–16]. CKD manifests in childhood in half of the patients: 47% in a French cohort (n = 30, median age at onset of CKD 6.5 years, range 1.5–18.6 years) [5], 43% in a multicenter European cohort (n = 83, 7 to 33 years) [9], and 50% in an American cohort (n = 50, median age at onset 11.9 years, 2.3 to 36.3 years) [14]. CKD was defined by an estimated glomerular filtration rate (GFR) below 80 mL/min/1.73 m^2^ in the French cohort [5], below 60 mL/min/1.73 m^2^ in the European and American cohorts [9, 14]. In all these studies, mut^0^ patients exhibited a higher frequency and a younger age at onset of CKD. Twelve to fourteen percent of patients evolve to end-stage renal disease (ESRD) requiring renal replacement therapy. In the French cohort, hemodialysis was started in 3 patients at the age of 5.9, 8.2 and 16 years [5]. The mechanisms responsible for renal failure in MMA remain poorly understood [9, 20–22]. Recently, an experimental study demonstrated a link between MMA, diseased mitochondria, mitophagy dysfunction and epithelial stress in tubular renal cells [23]. However, few observations report proximal tubulopathy or distal tubular acidosis type 2 [24, 25].

Our main objective in the present study was to precisely address the clinical and biological characteristics of long-term CKD in MMA adolescent and adult patients. To this aim, we investigated tubular functions, focusing on proximal tubular function. We also assessed the long-term renal function and we compared measured GFR (mGFR) by iohexol clearance to estimated GFR (eGFR) by Schwartz formula and CKD-EPI.

## Patients and methods

### Patients

**Fig. 1:**
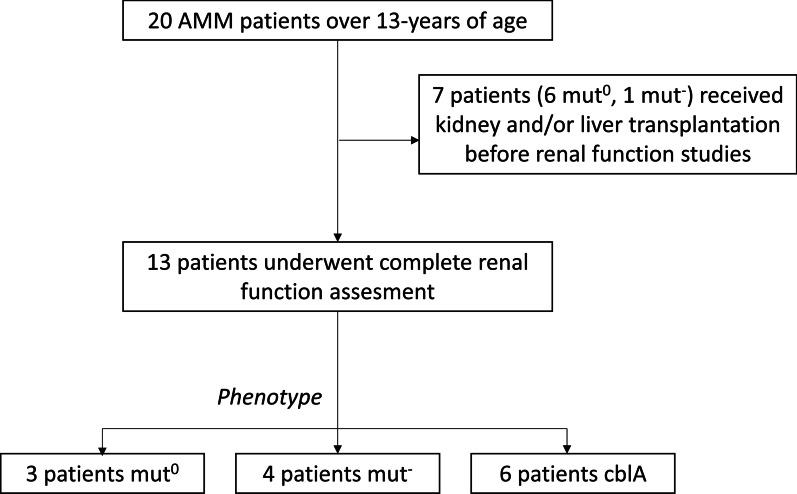
Flow chart

Twenty MMA patients older than 13 years of age were followed at Necker-Enfants Malades hospital, a French and European reference center for inborn metabolism diseases (MetabERN), between 2017 and 2018. Seven patients (6 mut^0^, 1 mut^−^) had received kidney and/or liver transplantation before renal function studies and were excluded from the present study. We retrospectively included all 13 remaining MMA patients (Fig. 1). Renal function was measured in these patients during routine follow-up. This study was performed in accordance with the ethical standards of the Helsinki Declaration. All patients and/or their legal tutors provided authorization for the use of their information for research purposes.

### Blood and urine biochemical tests

Biochemical tests were performed on blood and urine samples concurrently with the renal function studies. Methylmalonic acid levels were determined by isotope dilution gas chromatography – mass spectrometry. Creatinine was measured using IDMS-traceable enzymatic measurement.

### Measure of GFR by iohexol clearance (mGFR)

A direct intravenous injection of 300 mg of iohexol (OMNIPAQUE) was performed. Thereafter, plasma was harvested every hour for 5 h for determination of iohexol concentration by HPLC. Patients were asked to drink 400 mL of water for 30 min after injection of iohexol, then 150 mL per hour from the second hour to the end of the test. Iohexol clearance was used to define CKD stage according to Kidney Disease Outcomes Quality Initiative (KDIGO) CKD classification [26].

### Estimated GFR (eGFR)

We used simplified Schwartz formula to calculate eGFR [27]. In the patients older than 18 years of age, we also used the CKD-EPI formula [28].

### Ultrasonography measurements

Abdominal ultrasound studies were performed in all patients except one (#05) at the time of renal function studies. Renal length was measured in the longitudinal axis and compared with renal length nomograms developed for left and right kidneys separately, height being the independent variable [29].

### Statistical analysis

Descriptive statistical methods (medians and ranges) were used to assess the distributions of variables. Mann–Whitney test for continuous variables and Fisher’s exact tests for categorical variables were performed. Correlations between quantitative variables were assessed with Pearson product-moment correlation coefficient. The Bland–Altman method was used for assessing agreement between mGFR and eGFR. For all analyses, a p value < 0.05 was considered as significant. All analyzes were performed using InStat 3 software (GraphPad Software, San Diego, CA) and Prism 4 (GraphPad Software).

## Results

### Patients

Thirteen patients (F/M = 5/8) were included (Table 1). The median age at the time of the study was 24 years (13 to 32 years). Four patients had a neonatal onset disease (4 to 17 days) whereas 9 had a later onset disease (median age at diagnosis 6 months, 3 to 168 months). Patients were included in the following biochemical groups: mut^0^ (n = 3), mut^−^ (n = 4) and cblA (n = 6). Six patients had a B12-responsive disease (5 cblA and 1 mut^−^).

**Table 1:** Patients’ characteristics

Patient	Sex	Age at diagnosis	Gene	Mutation	Biochemical phenotype	B12-responsive disease	Past medical history	Age (years) at time of renal analysis
#01	F	Birth	*MUT*	A731T/A731T	mut^0^	No	Vesicoureteral reflux	17
#02	M	3.5 years	*MUT*	N219Y/Q383H	mut^0^	No	Left hydronephrosis on junction syndrome, optic atrophy	20
#03	F	Birth	*MUT*	R511X/G642R	mut^0^	No	Hypokinetic cardiac disease	23
#04	F	8 months	*MUT*	G203R/M740K	mut^−^	No	Hypothyroidism; viral myocarditis	28
#05	M	Birth	*MUT*	S342X/R694W	mut^−^	No		22
#06	F	3 months	ND	ND	mut^−^	Yes	Spine fractures	24
#07	F	2.5 years	ND	ND	mut^−^	No	Asthma	32
#08	M	2 years	ND	ND	cblA	Yes	Anorexia during late childhood	25
#09	M	6 months	*MMAA*	R22X/R22X	cblA	No	2 fractures of the right arm	16
#10	M	14 years	ND	ND	cblA	Yes	Single pelvic kidney	32
#11	M	3 months	*MMAA*	R145X/R145X	cblA	Yes	Obstructive and restrictive lung disease, osteoporosis	30
#12	M	8 months	*MMAA*	K276N/K276N	cblA	Yes	Osteoporosis	27
#13	M	birth	ND	ND	cblA	Yes		13

M, male; F, female; ND, not determined

At the time of the study, median protein intake was 35 g/day (22 to 46 g/day). We found no association between protein intake, B12-responsive disease, MMA phenotype and age. Median plasmatic MMA was 128 µmol/L (30 to 1139 µmol/L; normal value < 0.4 µmol/L) and median urinary MMA was 914 μmol/mmol (173 to 5619 μmol/mmol; normal value < 2 µmol/mmol).

### Renal morphology

Three out of 13 patients had kidney abnormalities belonging to the spectrum of Congenital Abnormalities of the Kidney and Urinary Tract (CAKUT): vesicoureteral reflux (Patient #01), left hydronephrosis on junction syndrome (#02), single pelvic kidney (#06).

Using ultrasound examination, median renal length was 101 mm (range 87 to 119 mm). Length data correspond to an average for both kidneys of each patients. The size difference of the two kidneys was less than 10 mm for all patient except one (#02), due to left hydronephrosis on junction syndrome. In the remaining 11 patients, the median size difference between the two kidneys was 5 mm (range: 1 to 9 mm). The median difference between measured and expected renal length was − 1.3 mm (range − 19 to + 12 mm; *p* = 0.73). Seven out of 12 MMA patients studied for kidney length had a diminished renal length compared to published nomograms [29]. Renal length was significantly lower than expected in cblA patients (median: − 9 mm, range − 19 to 1 mm, *p* = 0.03).

### mGFR by iohexol clearance

**Fig. 2:**
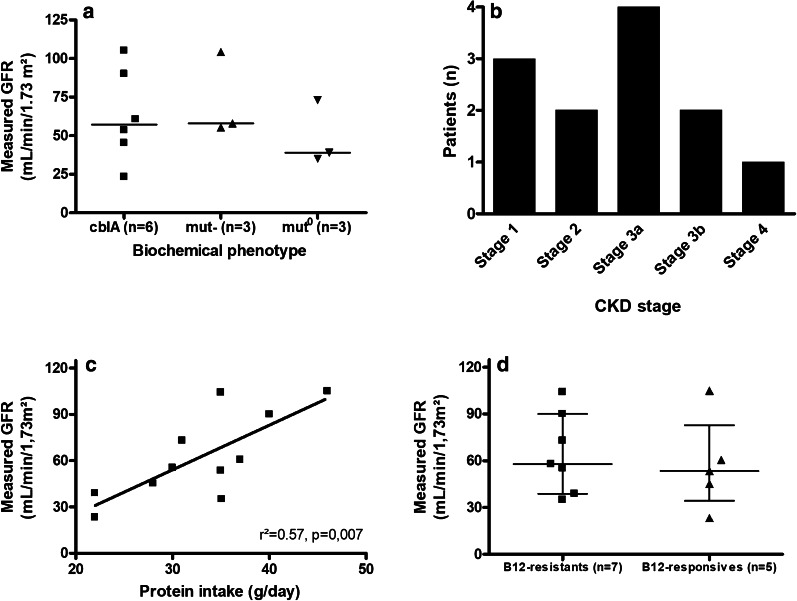
Measured GFR by clearance of iohexol. **a** Measured GFR (mL/min/1.73 m^2^) according to biochemical phenotype. Medians. **b** Patients (n) by CKD stage according to KDIGO stages: stage 1, GFR > 90 mL/min/1.73 m^2^; stage 2, GFR between 60 and 90 mL/min/1.73 m^2^; stage 3a, GFR between 45 and 60 mL/min/1.73 m^2^; stage 3b, GFR between 30 and 45 mL/min/1.73 m^2^; stage 4, GFR between 30 and 45 mL/min/1.73 m^2^. **c** Measured GFR (mL/min/1.73 m^2^) according to protein intake (g/day). **d** Measured GFR (mL/min/1.73 m^2^) according to vitamin B12 responsiveness. Medians ± interquartile ranges. Abbreviations: CKD, chronic kidney disease; GFR, glomerular filtration rate

Measured GFR by iohexol clearance was obtained in 12 out of 13 patients (a technical problem occurred for patient #06 and its result was not interpretable). Median mGFR was 56.5 mL/min/1.73 m^2^ (23.3 to 105.0 mL/min/1.73 m^2^). Only 3 out of 12 patients (2 cblA and 1 mut-) had normal renal function (Fig. 2a). Two patients had CKD stage 2, 4 had CKD stage 3a, 2 had CKD stage 3b and 1 had CKD stage 4 (Fig. 2b). Measured GFR was significantly associated with protein intake (R = 0.84 [0.45; 0.96], *p* = 0.002). We did not find any association between mGFR, vitamin B12-responsive disease, MMA phenotype, age, plasma and urinary AMM concentrations (Fig. 2a, c, d).

### eGFR

**Fig. 3:**
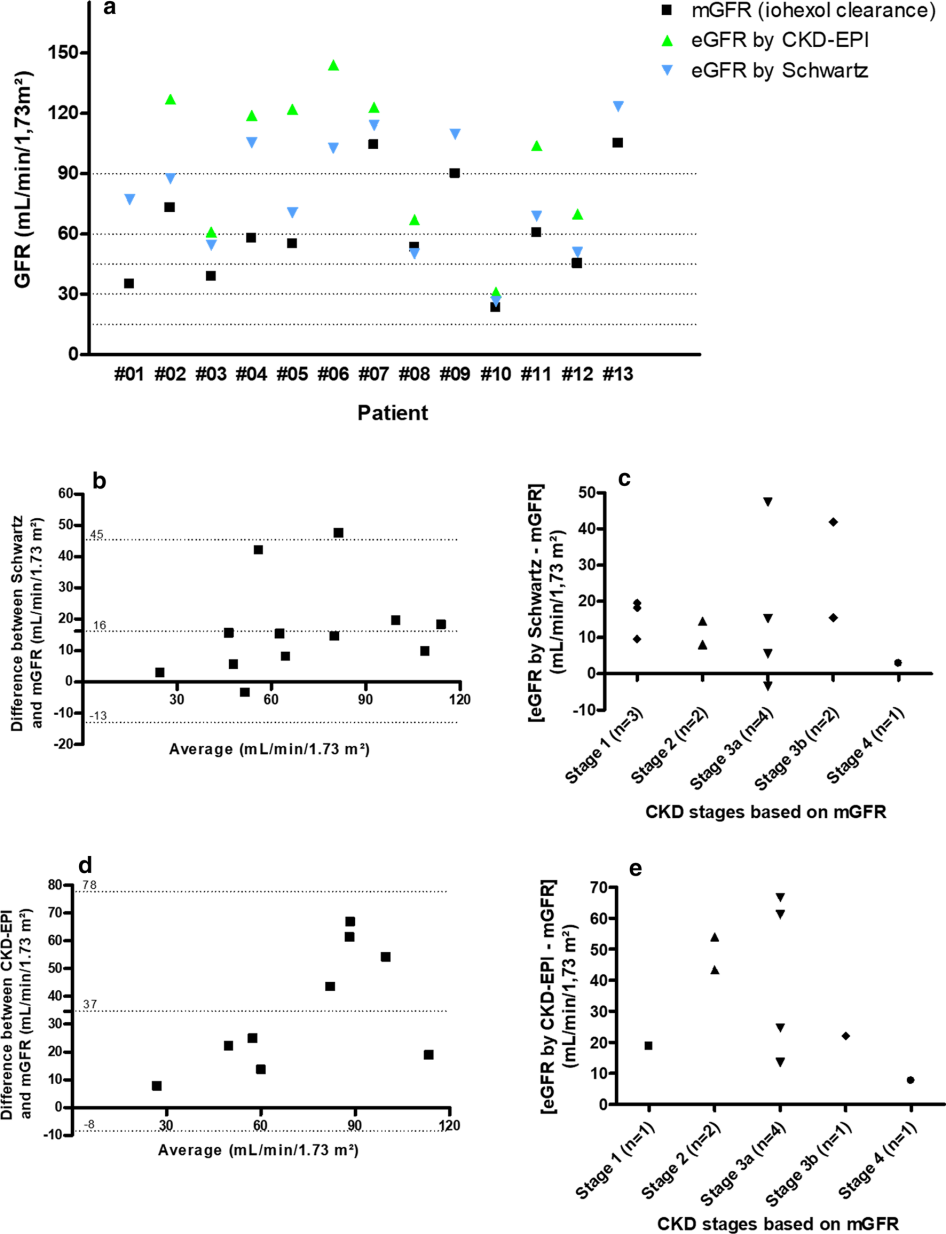
The usual formulas for estimated GFR overestimated the renal function of MMA patients. **a** Measured GFR by clearance of iohexol, Estimated GFRs by Schwartz formula and by CKD-EPI (mL/min/1,73m^2^). **b** Schwartz formula overestimated mGFR with bias 16 ± 15 mL/min/1.73, 95%LOA [− 13 to 45], Bland–Altman method. **c** Difference between eGFR by CKD-EPI and mGFR according to CKD stages. **d** CKD-EPI overestimated mGFR with bias 37 ± 22 mL/min/1.73, 95%LOA [− 8 to 78], Bland–Altman method. **e** Difference between eGFR by Schwartz formula and mGFR according to CKD stages. CKD, chronic kidney disease; GFR, glomerular filtration rate; eGFR, estimated GFR; LOA, limit of agreement; mGFR, measured GFR

Median eGFR by Schwartz formula was 77 mL/min/1.73 m^2^ (26 to 123 mL/min/1.73 m^2^). Schwartz formula significantly overestimated GFR compared to mGFR: + 16 ± 15 mL/min/1.73 m^2^, 95% limit of agreement (LOA) [− 13 to 45 mL/min/1.73 m^2^] (Fig. 3a, b). Four out of 12 patients were misclassified in the KDIGO CKD classification when the Schwartz formula was used (Fig. 3c). Ten patients were over 18 years of age, allowing calculating eGFR by CKD-EPI. CKD-EPI systematically overestimated GFR compared to mGFR: + 37 ± 22 mL/min/1.73, 95% LOA [− 8 to 78 mL/min/1.73 m^2^] (Fig. 3a, d). Only one patient was properly classified when the CKD-EPI was used for the KDIGO CKD classification (Fig. 3e).

### Investigation of tubular function

#### Potassium

**Fig. 4:**
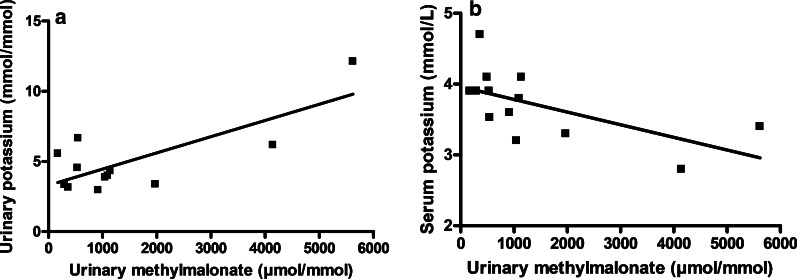
Hypokalemia was due to a nonreabsorbable anion effect of methylmalonate. **a** Urinary potassium (mmol/mmol of creatininuria) was positively associated with urinary methylmalonate (µmol/mmol of creatininuria), R = 0.77, *p* = 0.004. **b** Serum potassium (mmol/L) was negatively associated with urinary methylmalonate (µmol/mmol of creatininuria), R = − 0.61, *p* = 0.03

Median serum potassium level was 3.8 mmol/L (2.8 to 4.7 mmol/L). Serum potassium was significantly lower in mut^−^ patients (3.3 mmol/L, 2.8 to 3.6 mmol/L) than in mut^0^ (4.1 mmol/L, 3.7 to 4.1 mmol/L, *p* = 0.05) and cblA patients (3.9 mmol/L, 3.5 to 4.7 mmol/L, *p* = 0.02) (Additional file 2: Fig. S2). Three out of 13 patients (#04, #05 and #07), all being mut-, had hypokalemia < 3.5 mmol/L. None of these patients had high blood pressure. A mut^0^ patient (#13) had plasma potassium at 3.7 mmol/L despite potassium replacement therapy (3.6 g/day) and CKD stage 3b (Table 2). Patients #03, #04 and #05 had inadequate urinary potassium level. Urinary potassium and urinary methylmalonate were strongly correlated (R = 0.77, *p* = 0.004) (Fig. 4a). Conversely, we found a negative correlation between serum potassium and urinary methylmalonate (R = − 0.61, *p* = 0.03) (Fig. 4b). Taken together, these results suggest a non-reabsorbable anion effects of urinary methylmalonate that increases kaliuresis. There was no correlation between mGFR and serum potassium.

**Table 2:** Biological results

	Median	Min–Max	Standards
Measured GFR (mL/min/1.73 m^2^)	56.5	23–105	> 90
Estimated GFR by Schwartz formula (mL/min/1.73 m^2^)	77	26–123	> 90
Estimated GFR by CKD-EPI (mL/min/1.73 m^2^)	112	31–144	> 90
*Serum laboratory data*			
Urea (mmol/L)	3.8	2.4–8.1	2.5–8.0
Sodium (mmol/L)	138	135–141	136–146
Potassium (mmol/L)	3.8	2.8–4.7	3.5–4.5
Chloride (mmol/L)	105	100–108	98–107
Uric acid (µmol/L)	421	202–703	150–350
Magnesium (mmol/L)	0.83	0.68–0.96	0.85–1.15
Alkaline reserve (mmol/L)	25	22.3–30	22–29
Total calcium (mmol/L)	2.3	2.2–2.6	2.25–2.6
Ionized calcium (mmol/L)	1.17	1.13–1.24	1.15–1.34
Phosphorus (mmol/L)	1.0	0.79–1.4	0.85–1.5
PTH (pg/mL)	47.2	28.9–106.6	10–50
25OH vitamin D (ng/mL)	28	15–76	30–80
1–25 OH vitamin D (pg/mL)	43	23–106	30–60
*Urinary parameters*			
Fractional excretion of sodium (%)	1.1	0.2–7.0	NA
Potassium (mmol/L)	47.5	13.4–118	NA
Fractional excretion of potassium (%)	20.8	5.7–63.9	NA
Calcium (mmol/L)	0.5	0.5–2.77	< 3.6
Calcium (mmol/mmol)**	0.07	0.02–0.28	< 0.55
Fractional excretion of calcium (%)	1.8	0.5–4.1	NA
Fractional excretion of urea (%)	5.1	0.04–12.2	NA
Fractional excretion of phosphate (%)	9.6	1.5–48.6	NA
Fractional excretion of magnesium (%)	4.9	2.0–11.3	NA
TmP-GFR (mmol/L)*	0.9	0.5–1.4	0.7–1.4
Proteinuria (mg/mmol)**	8.5	0–34.6	< 50
Microalbuminuria (mg/mmol)**	0.8	0–15.4	< 35
β2-microglobulinuria (µg/mmol)**	6.0	0–506	< 35

GFR, glomerular filtration rate; NA, non-applicable; TmP-GFR, tubular maximum reabsorption capacity of phosphate

*Urinary analytes per mmol of urinary creatinine

**mGFR was used to calculate TmP-GFR

#### Acid–base status

The biological results are summarized in Table 3. All patients had normal alkaline with median alkaline reserve 25 mmol/L (22.3 to 30 mmol/L). Only one patient (#03) received sodium bicarbonate replacement therapy (9 g/day).

**Table 3:** Characteristics of the 4 patients having urinary loss of potassium

Patient	Measured GFR (mL/min/1.73 m^2^)	Kalemia (mmol/L)	Urinary potassium (mmol/L)	Fractional excretion of potassium (%)
#03	38.9	3.7*	64	64
#04	57.8	2.8**	45	6
#05	55.3	3.3	51	28
#07	104.2	3.2	13	15

GFR, glomerular filtration rate

*Potassium replacement therapy: 3600 mg/day

**Potassium replacement therapy: 1200 mg/day

#### Uric acid

None of the patients had past history of stones or gout. None of the patients had hypouricemia suggestive of proximal tubulopathy. As expected, serum uric acid tended to be inversely proportional to mGFR (*p* = 0.054).

#### Phosphocalcic metabolism

Even if all patients received 25OH vitamin D replacement therapy, median 25OH vitamin D was 28 ng/ mL (15 to 76 ng/ mL). Seven out of 13 patients (54%) had 25OH vitamin D deficiency below 30 ng/mL. Median 1-25OH vitamin D was 43 pg/mL (23 to 106 pg/mL). Two patients had 1–25 OH vitamin D deficiency below 30 pg/mL, one of them (#08) having both 25OH and 1-25OH deficiency. Median PTH was 47.2 pg/mL (28.9 to 106.6 pg/ mL, normal value 10–50 pg/mL). Six out of 13 patients (46%) had high PTH level above 50 pg/mL. Hyperparathyroidism was secondary to 25OH vitamin D deficiency in 2 patients and was associated with mGFR below 45 mL/min/1.73 m^2^ in 4 patients. Two out of 13 patients received calcium replacement therapy. Median ionized calcemia was 1.17 mmol/ L (1.13 to 1.24 mmol/L). Two patients (#07 and #08) had a slight decrease of ionized calcemia below 1.15 mmol/ L, related to a 25OH vitamin D deficiency, with adequate calciuria. Median phosphatemia was 1.0 mmol/L (0.79 to 1.4 mmol/L). Two patients (#05 and #06) had hypophosphatemia below 0.85 mmol/L without renal phosphate leakage, as indicated by normal tubular maximum reabsorption capacity of phosphate (TmP-GFR).

#### Magnesium

Median magnesium in plasma was 0.83 mmol/L (from 0.68 to 0.96 mmol/L). Three out of 13 patients (#06, #07 and #10) had hypomagnesemia below 0.75 mmol/L. Patient #10 had a high urinary fractional excretion of the magnesium (11.3%), suggesting a renal loss of magnesium. Patients #06 and #07 had suitable fractional excretion of the magnesium.

#### Urinary markers of tubular dysfunction

No patient had glycosuria. Only one patient, Patient #10, had a marked elevation of β2-microglobulinuria (506 μg/mmol), suggesting proximal tubulopathy. Interestingly, he also had the more severe CKD, defined by mGFR of 23.3 mL/min/1.73m^2^. The other patients did not display any low molecular weight proteinuria.

To conclude, no patient had complete tubular proximal syndrome. Only one patient (#10) had biological signs suggestive of incomplete proximal tubulopathy with both elevation of β2-microglobulinuria and renal loss of magnesium. Four out of 13 patients had isolated potassium loss related to a non-reabsorbable anion effect.

### Glomerular dysfunction markers

No patient had microscopic hematuria or proteinuria above 500 mg/g. Median albumin to creatinine ratio was low 0.8 mg/mmol (0 to 15.4 mg/mmol). Three out of 13 patients (#01, #05 and #08) display albuminuria above 3 mg/mmol.

## Discussion

CKD is a common complication of MMA and worsens long-term prognosis. We precisely studied the renal function of 13 adolescent and adult MMA patients who had not received a liver and/or kidney graft. Our study confirms the high prevalence of CKD in MMA patients: median mGFR was 56.5 mL/min/1.73 m^2^, 77% of patients had a mGFR below 90 mL/min/1.73 m^2^, more than half (54%) had at least moderate renal impairment (mGFR below 60 mL/min/1.73 m^2^), and one had severe renal impairment (mGFR below 15 mL/min/1.73 m^2^) [5, 9, 14]. Surprisingly, mGFR and vitamin B12-responsiveness were not correlated, even if vitamin B12-responsive patients are considered less severe. Similarly, we did not find any correlation between biochemical phenotype and mGFR. This result contrasts with the expectation that renal function would be poorer in mut^0^ patients but is likely due to biased sampling of mut^0^ patients in our study. Indeed, 6 out of 9 MMA patients over 13-years of age followed in our hospital were not included in the present study because they previously received a liver and/or kidney graft (Fig. 1). Furthermore, 6 out of 7 patients that received kidney and/or liver graft before this study were mut^0^, highlighting the high prevalence of ESRD in mut^0^ patients. Two out of 3 mut^0^ patients included in the present study received combined liver and kidney transplantation in the year following the study; the discussion is ongoing for the third patient. Interestingly, protein intake showed a strong positive correlation with eGFR. As protein intake is finely adjusted by dietary management according to metabolic balance, this result suggests a major role of the renal parenchyma in the metabolic balance of MMA. Brassier et al. [12] previously reported metabolic improvement in 4 mut^0^ patients who received a renal graft alone (without liver graft), which reinforces this hypothesis. After renal transplantation, the number of decompensations per patient per year decreased and the protein intake significantly increased [12]. Whereas liver transplantation, with or without kidney transplantation according to GFR, remains the gold standard to improve the quality of life, the neuropsychological development and the metabolic balance when facing chronic metabolic decompensation, an isolated kidney transplantation could also be individually discussed [30].

It has been suggested that CKD in MMA is the consequence of a tubular dysfunction. No case of glomerulopathy has been reported in the literature and our study supports this data. When performed, renal biopsy showed severe interstitial fibrosis and tubular atrophy [5, 21, 24, 31], with ultrastructural (enlarged mitochondria in proximal tubules) and functional (loss of cytochrome C, decrease in NADPH activity) alterations. These histological findings are consistent with experimental studies suggesting that CKD could be the consequence of mitochondrial dysfunction in proximal tubule [15, 21, 22].

The hypothesis of a chronic tubulopathy is supported by experimental studies [15, 21, 22]. In a murine model of MMA renal disease, mice developed chronic tubulointerstitial nephritis and a decreased GFR associated with megamitochondria formation in the proximal tubules [21, 32, 33]. Targeting mitochondrial dysfunction by administration of coenzyme Q10 and vitamin E, the authors demonstrated that antioxidants attenuate the renal disease induced by high protein diet in a murine model of MMA [21]. In an in vitro model of tubular epithelial cells isolated from urines of MMA patients, Ruppert et *al.* showed not only a disturbance of energy metabolism in glycolysis, mitochondrial respiratory chain and Krebs cycle but also increased reactive oxygen species formation [22]. Recently, a study demonstrated that metabolic and mitochondrial alterations are exacerbated by anomalies in PINK1/Parking-mitophagy, providing new therapeutic perspectives for MMA [23]. However, few clinical observations support the hypothesis that CKD could be due to a tubulopathy associated with mitochondrial dysfunction [24, 25]. In our study, we precisely characterized tubular functions of MMA patients. No patient had complete tubular proximal syndrome. Only one patient had biological signs suggestive of incomplete proximal tubulopathy with both elevation of β2-microglobulinuria and renal loss of magnesium, but it was associated with severe renal failure. Four out of 13 patients had isolated potassium loss, probably due to a non-reabsorbable anion effect of urinary methylmalonate. Methylmalonate acts as nonreabsorbable anions, enhancing potassium excretion by increasing transtubular potential difference. Such non-reabsorbable anion effect has long been well described with penicillins, also acting as non-reabsorbable anion [34, 35]. Indeed, we found a positive correlation between urinary potassium and urinary methylmalonate as well as a negative correlation between serum potassium and urinary methylmalonate. In clinical practice, patients may experience aggravation of renal function during acute metabolic decompensations, which could partially be explained by toxicity of MMA on renal cells [21, 22, 36]. Moreover, the renal evolution in MMA is completely different from that observed in classical causes of hereditary tubulopathies such as cystinosis or Lowe syndrome. In cystinosis patients, the most common cause of hereditary tubulopathy in pediatrics, severe Fanconi syndrome precedes CKD and ESRD [37]. Lithiasis and nephrocalcinosis are classical features of Lowe syndrome but were not observed in our cohort of MMA patients [38]. Gitelman syndrome, the most common cause of hereditary distal tubular diseases, is also different, associated with profound hypokalemia but normal renal function [39]. Taking together, without excluding involvement of mitochondrial dysfunction in the proximal tubule, these observations suggest a preponderant interstitial origin of CKD in MMA. A better understanding of the mechanisms responsible for CKD in MMA is mandatory to improve the care of these patients.

Furthermore, as expected, eGFR significantly overestimates renal function in MMA patients with low protein diet and reduced muscle mass: + 16 ± 15 mL/min/1.73 m^2^ with Schwartz formula and + 37 ± 22 mL/min/1.73 m^2^ with CKD-EPI [40]. Indeed, both formulas are partly based on evaluation of skeletal muscle mass, which is dramatically decreased in MMA patients. Respectively 25% and 90% of MMA patients were misclassified when the Schwartz formula and CKD-EPI formulas were used to categorize subjects according to the KDIGO CKD classification. Therefore, mGFR is essential and should be systematically performed when therapeutic decisions such as dialysis or transplantation are discussed. Schwartz formula was significantly better than CKD-EPI to estimate GFR and had to be preferred if mGFR cannot be performed.

To conclude, CKD is a common complication of the MMA, which worsens long-term prognosis. Mild evidence of proximal tubular dysfunction was found in only one out of 13 patients, suggesting the existence of other mechanisms responsible for CKD during MMA. A better understanding of the mechanisms responsible for CKD in MMA is mandatory to improve the care of these patients.

## Supplementary Information

**Additional file 1: Fig. S1**. Methylmalonic acidemia is caused by a defect in the mitochondrial methylmalonyl-CoA mutase (MCM). MCM isomerises L-methylmalonyl-CoA into succinyl-CoA which enters the Krebs cycle. Its cofactor is adenosylcobalamin. The MUT, MMAA and MMAB genes respectively encode for MCM, CblA and CblB. MCM deficiencies are due to mutations in the MUT gene or to mutations in MMAA or MMAB. MCM deficiency results in accumulation of toxic metabolites such as 3-hydroxypropionic acid, methylcitric acid and most markedly, methylmalonic acid.

**Additional file 2: Fig. S2**. Serum potassium was lower in patients with mut- phenotype. Medians ± interquartile ranges.

## Data Availability

The data that support the findings of this study are available from the corresponding author upon reasonable request.

## Abbreviations

CAKUT: Congenital abnormalities of the kidney and the urinary tract
CKD: Chronic kidney disease
eGFR: Estimated glomerular filtration rate
ESRD: End-stage renal disease
GFR: Glomerular filtration rate
KDIGO: Kidney Disease Outcomes Quality Initiative
MCM: Methylmalonyl-CoA mutase
mGFR: Measured glomerular filtration rate
MMA: Methylmalonic acidemia
