# Supplementary figures and images for: Long-term renal outcome in methylmalonic acidemia in adolescents and adults
Source: Orphanet J Rare Dis. 2021 May 13;16:220. doi: 10.1186/s13023-021-01851-z (PMC8120835; doi:10.1186/s13023-021-01851-z)

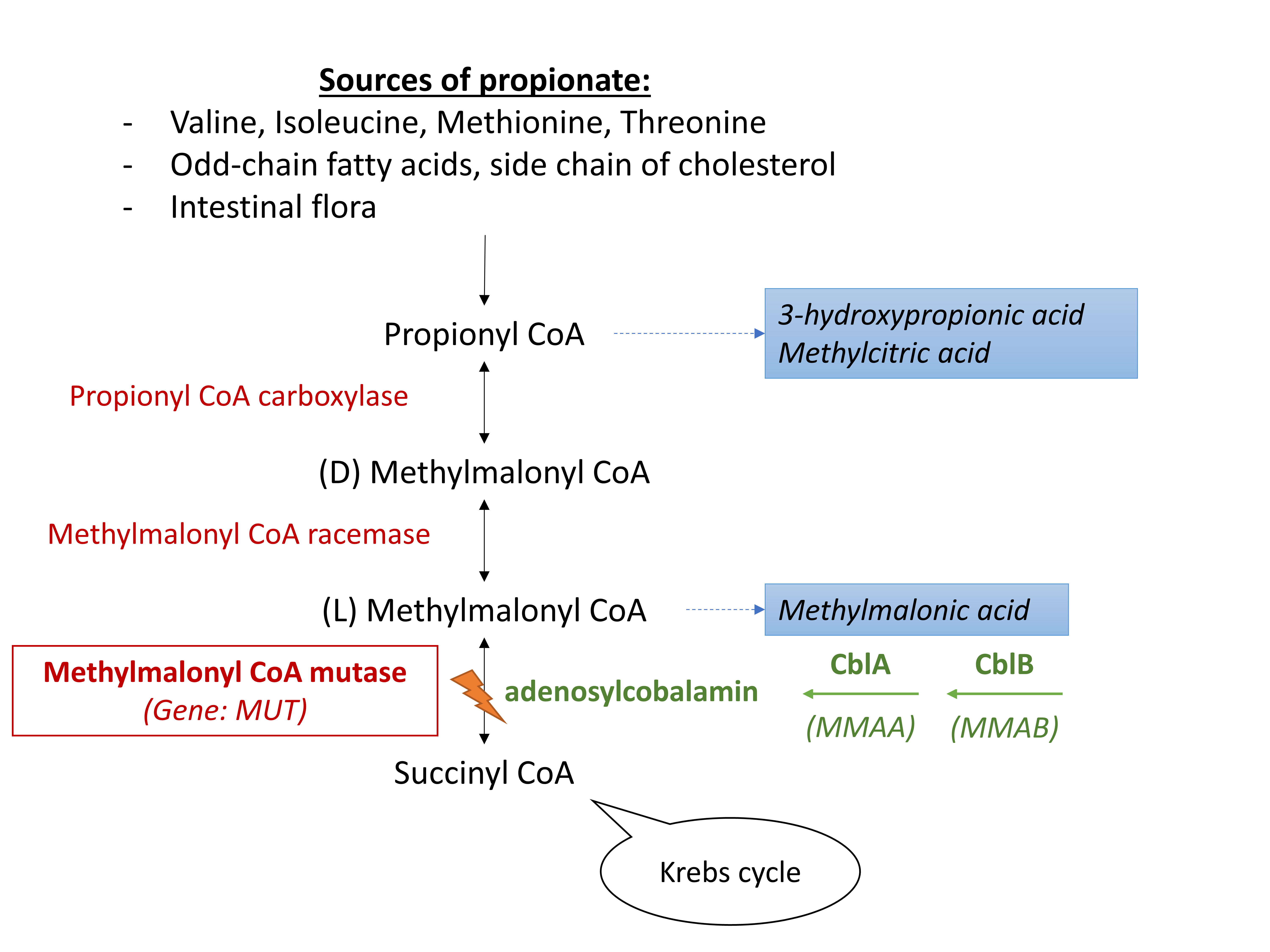

Supplement: Supplementary file 1 — Additional file 1: Fig. S1. Methylmalonic acidemia is caused by a defect in the mitochondrial methylmalonyl-CoA mutase (MCM). MCM isomerises L-methylmalonyl-CoA into succinyl-CoA which enters the Krebs cycle. Its cofactor is adenosylcobalamin. The MUT, MMAA and MMAB genes respectively encode for MCM, CblA and CblB. MCM deficiencies are due to mutations in the MUT gene or to mutations in MMAA or MMAB. MCM deficiency results in accumulation of toxic metabolites such as 3-hydroxypropionic acid, methylcitric acid and most markedly, methylmalonic acid. [file 13023_2021_1851_MOESM1_ESM.tif]

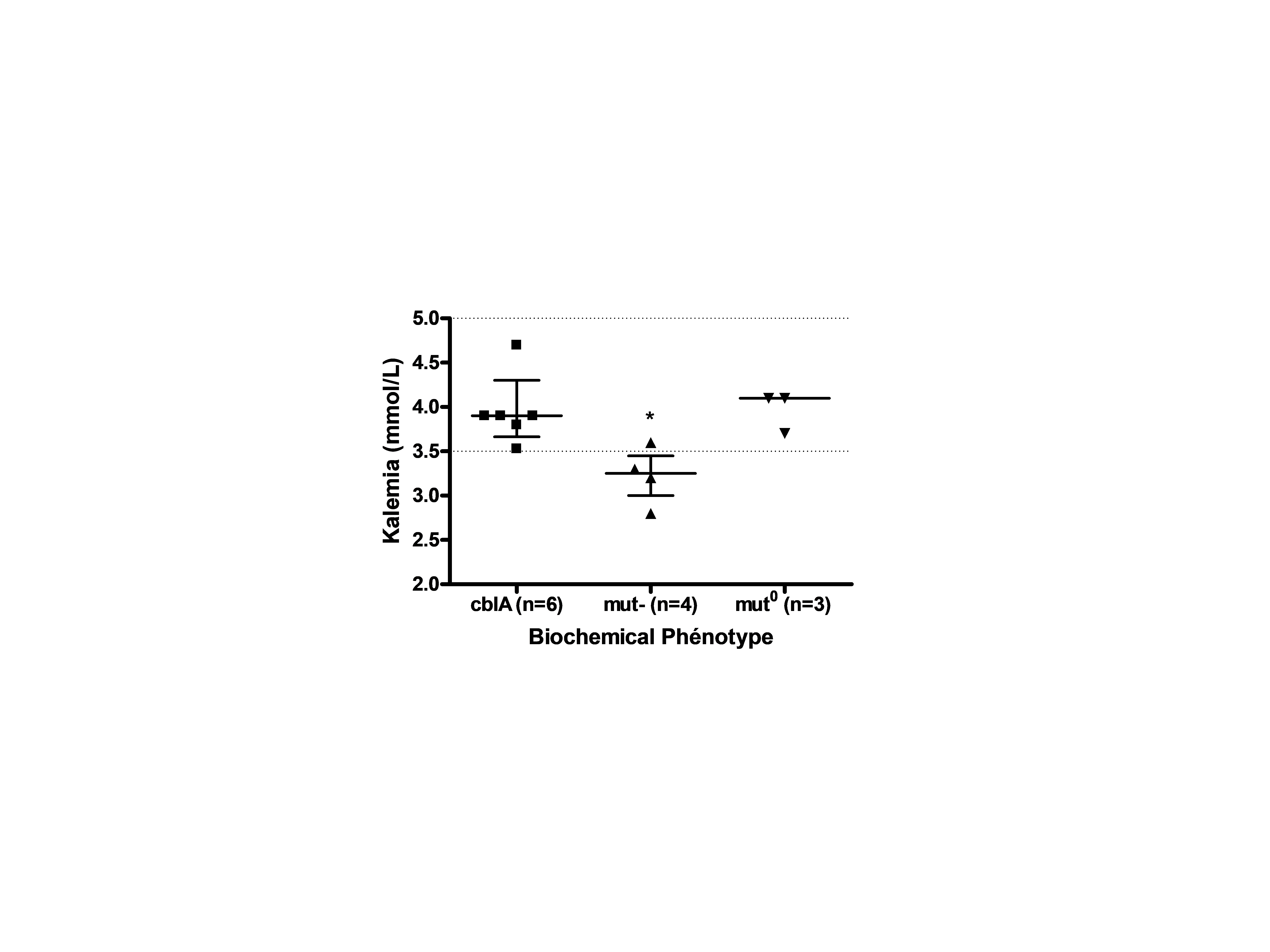

Supplement: Supplementary file 2 — Additional file 2: Fig. S2. Serum potassium was lower in patients with mut- phenotype. Medians ± interquartile ranges. [file 13023_2021_1851_MOESM2_ESM.tif]
